# Loss-tolerant measurement-device-independent quantum private queries

**DOI:** 10.1038/srep39733

**Published:** 2017-01-04

**Authors:** Liang-Yuan Zhao, Zhen-Qiang Yin, Wei Chen, Yong-Jun Qian, Chun-Mei Zhang, Guang-Can Guo, Zheng-Fu Han

**Affiliations:** 1 Key Laboratory of Quantum Information, University of Science and Technology of China, CAS, Hefei, 230026, China; 2 Synergetic Innovation Center of Quantum Information & Quantum Physics, University of Science and Technology of China, Hefei, 230026, China

## Abstract

Quantum private queries (QPQ) is an important cryptography protocol aiming to protect both the user’s and database’s privacy when the database is queried privately. Recently, a variety of practical QPQ protocols based on quantum key distribution (QKD) have been proposed. However, for QKD-based QPQ the user’s imperfect detectors can be subjected to some detector- side-channel attacks launched by the dishonest owner of the database. Here, we present a simple example that shows how the detector-blinding attack can damage the security of QKD-based QPQ completely. To remove all the known and unknown detector side channels, we propose a solution of measurement-device-independent QPQ (MDI-QPQ) with single- photon sources. The security of the proposed protocol has been analyzed under some typical attacks. Moreover, we prove that its security is completely loss independent. The results show that practical QPQ will remain the same degree of privacy as before even with seriously uncharacterized detectors.

An ideal symmetrically private information retrieval (SPIR) protocol^1^ allows a user, e.g. Alice, to extract an item of a database without revealing any information about which one she has retrieved to the database owner, e.g. Bob (*perfect user privacy*). Meanwhile, Alice can obtain only one item in a single query (*perfect database privacy*). SPIR can be used in the internet search and online transactions for the valuable and sensitive information. A SPIR protocol is a 1-out-of-N oblivious transfer (OT) protocol essentially^2^. In the 1-out-of-N OT, Bob sends *N* bits and Alice chooses which one she obtains. At the end of the protocol Alice knows the chosen bit value but has no information about other bits, while Bob is entirely ignorant of which bit Alice received. The security of classical OT relies on the unproven computational assumptions^1^. Unfortunately, Lo has proven that quantum mechanics along cannot provide unconditionally secure perfect quantum OT either^3^. This implies the impossibility of perfect quantum SPIR. It can be concluded from Lo’s proof that if a quantum SPIR has perfect user privacy, then Alice can perform an Einstein-Podolsky-Rosen-type^4^ attack to access the entire database without being detected.

Despite the no-go theorem about ideal quantum SPIR, some interesting degree of security can be achieved with changes in the model or the security requirements of the protocol. The first attempt of combining the quantum mechanics with SPIR was made by Kerenidis and De Wolf 5. However, in their protocol, the database is replicated over more than one owner and it preserves database privacy against only honest user. In 2008, Giovannetti, Lloyd and Maccone proposed a cheat sensitive quantum protocol (GLM08 protocol), named quantum private queries (QPQ), to solve the SPIR problem^6^. The term cheat sensitive means that Alice can catch Bob cheating with a nonvanishing (but nonunity) probability if Bob attempts to learn what Alice queries. The imperfect user privacy is the reason that QPQ can evade the no-go proof of Lo^3^. The security of GLM08 has been analyzed strictly^7^ and a proof-of-principle experiment has been implemented by De Martini *et al*.^8^. In the experiment, the bits of database were represented by an array of half-wave plates. If there existed a half-wave plate in one spatial mode, it meant that the corresponding bit was 1. Otherwise the bit was 0. For the user Alice, she prepared two non-orthogonal polarized states (the query state and test state), and sent them to Bob in a random order. The query state was routed into the desired spatial mode to obtain the retrieved bit value. Combining this value with the test state, Alice could verify the honesty of Bob with a certain probability.

The advantage of GLM08 and its improved version^9^ is that the communication and computational complexity has been reduced exponentially. However, the security of the protocols may be seriously compromised in the presence of losses and it will be difficult to retrieve when the dimension of the database is large. In 2011, Jakobi *et al*.^10^ proposed a QPQ protocol (J^+^11 protocol) based on the Scarani-Acin-Ribordy-Gisin 2004 (SARG04) quantum key distribution (QKD) protocol^11^. J^+^11 is completely impervious to losses and can be easily implemented for large database with mature QKD technology. By adjusting the coefficients of the sent states, Gao *et al*. made the J^+^11 flexible for either better user privacy or better database privacy (G^+^12 protocol)^12^. Referring to the two-way QKD scheme, the QPQ has been designed to perform better in resisting the joint-measurement attack^13^. The QKD-based QPQ is a very practical solution and has been generalized with other QKD protocols^14^,^15^,^16^. The first experimental demonstration of J^+^11 and G^+^12 has been done on a QKD system^17^ with some necessary modifications by Chan *et al*.^18^. In their experiment, four polarized states from two orthonormal bases were prepared randomly by Bob using the phase-randomized weak coherent state (PR-WCS) source. The faint laser pulses were transmitted to Alice trough a 12.4 km dark fiber with sequences of strong light, which acted as quantum frames^17^ to synchronize and compensate the time shift. Alice measured the faint pulses by passively selecting one of the bases randomly. After the classical postprocessing, including Bob announcing pairs of non-orthogonal states, key compression and error correction, Alice performed a total of 11 queries at the single-photon level. This experiment shows the feasibility of QKD-based QPQ with state-of-the-art technology. Note that the novel error-correcting code developed by Chan *et al*.^18^ and another one by Gao *et al*.^19^ to address the noise in the channel can protect the privacy of both parties. As the above QPQ protocols focused mainly on retrieving a single bit, multi-bit block QPQ has been proposed^20^,^21^, in which Alice could obtain several desired bits by just one query. Nevertheless, we still focus on the single-bit QPQ protocols in this paper for there are still many problems needed to be solved.

Note that the quantum processes of the J^+^11 have no difference from the implementation of SARG04 QKD. The practical security loopholes^22^,^23^, especially the detector side channels^24^,^25^,^26^, in QKD may still exist in practical J^+^11 system, which could damage the security of the system. In the following, we will give a strategy of detector-blinding attack on the practical J^+^11 and G^+^12 systems and show how it breaks the user privacy completely. To remove all the known and unknown detector-side-channel attacks launched by dishonest Bob, we propose a measurement-device-independent QPQ (MDI-QPQ) protocol with single-photon sources. MDI-QPQ benefits from the idea of MDI-QKD^27^,^28^,^29^,^30^,^31^,^32^,^33^,^34^,^35^,^36^ and enriches the application of the MDI paradigm in the mistrustful quantum cryptography^37^. Moreover, the security of our protocol is loss-tolerant.

In MDI-QKD, the detector side channels are removed by using the Bell state measurement (BSM) conducted by an untrusted third party. The two legitimate parties need only to know their state preparations. MDI-QPQ is similar to MDI-QKD in that Alice and Bob also need to characterize only their state prepare processes. A slight difference is that the BSM is played by Alice because there is no third party here. Nevertheless, Alice needs only to obtain a Bell state outcome from her measurement device, but does not have to characterize it anymore. Thus, the idea of MDI-QKD can be modified to defend a dishonest Bob’s detector-side-channel attacks in MDI-QPQ. However, note that the untrusted measurement device is now in Alice’s laboratory, which is different from MDI-QKD. We emphasize that Alice should protect the classical information of her state preparations from leakage to both the measurement device and Bob. Considering that, we must be careful when adapting the MDI paradigm into the mistrustful quantum cryptography where the legitimate parties do not trust each other.

## Results

### The principle of detector-blinding attack on the practical QKD-based QPQ systems

To demonstrate that the detector side channels do exit in previous practical QKD-based QPQ systems, let us take the detector-blinding attack as an example. We first briefly review the basic ingredients of the J^+^11 protocol. In the J^+^11 protocol, four qubits from two mutually unbiased bases, e.g., the rectilinear basis {|0〉, |1〉} and the diagonal basis {|+〉, |−〉}, are sent randomly from Bob to Alice. Here, |0〉 and |1〉 represent the horizontal and vertical polarization states, 
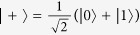
 and 
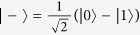
, respectively. The rectilinear basis stands for the raw key bit 0 and the diagonal basis refers to the raw key bit 1. Alice measures the states in the two bases randomly. For each of the successfully detected photons, Bob tells Alice two states which are composed of the actually sent state and a random one from the other basis. Combining with her measurement result, Alice can decode the raw key bit certainly with probability 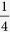. Then, the raw key are divided into several substrings by Bob with the length equaling to the database and are added bitwise to generate the final key. Ideally, Alice will know only one final key and Bob has no information of its position. Alice computes the position difference between the known final key and her desired element of the database, and announces it to Bob. Bob shifts the final key according to the difference value and encrypts the database. At last, Alice can obtain that element from the encrypted database privately with the known final key.

Seeing the quantum processes of J^+^11 are the same with SARG04 QKD, we find that the principle of the detector-blinding attack on practical J^+^11 system is similar to the one in QKD^26^,^38^ with changes only in the classical postprocesses. For definiteness, let Alice chooses the bases actively with two single-photon detectors. Assume that in classical linear mode, the detector *i* ∈ {1, 2} always clicks from a trigger pulse with optical peak power ≥ *p*_always,*i*_, and never clicks from a trigger pulse with optical peak power ≤ *p*_never,*i*_. A perfect detector-blinding attack is possible if the equation


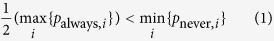


is satisfied^26^, where 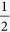 is the mutual projection probability of the honest states from different bases. Equation (1) implies that if Alice’s basis differs from Bob’s, then neither of the two detectors would click.

The procedures of the detector-blinding attack launched by dishonest Bob in practical J^+^11 system are as follows. First, Bob transmits bright light into Alice’s detectors to convert them into classical linear model. Then, instead of preparing the sent states in single-photon pulses, Bob sends them by bright trigger pulses with peak power just above 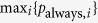. Consequently, Alice has a successful click only if her basis is consistent with the sent state due to equation (1). Meanwhile, the non-detected trials will be discarded and announced by Alice because of the loss-tolerant property of the protocol. Thus, Bob will know which cases Alice has successful clicks, and her bases and measurement results correspondingly. Based on these information, Bob can determine with certainty whether Alice will obtain a conclusive raw key bit. For example, Bob sends the state |0〉 and Alice has a click, which implies that Alice has chosen the rectilinear basis and obtained the |0〉 state. Then, Bob can make Alice acquire a conclusive raw key bit 1 if he announces the states pair {|1〉, |−〉}. Otherwise, he announces the states {|0〉, |+〉} and Alice will have an inconclusive result. Finally, Bob could control precisely which final key is known to Alice by dividing the raw key properly, which means the user privacy is damaged completely.

**Figure 1:**
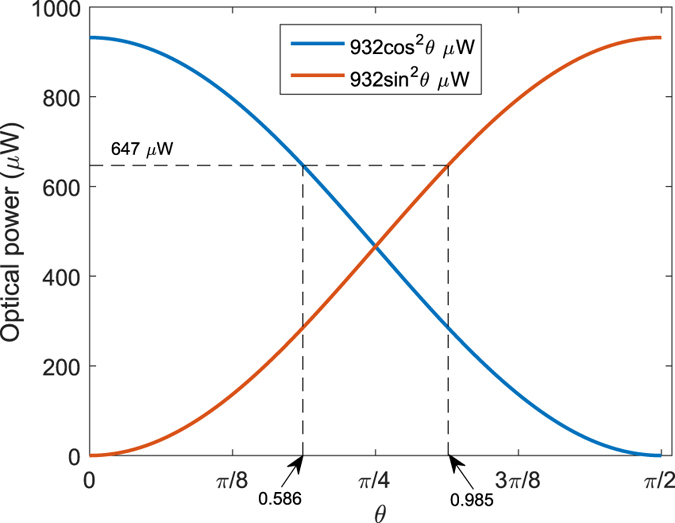
Relationship between the optical power of the pulse reaching the two detectors and the value of *θ* when Alice chooses a different basis with Bob. The dotted box indicates the range of *θ* that fulfils equation (2), which implies that dishonest Bob can launch a perfect detector-blinding attack.

As for G^+^12 protocol, Bob sends the states {|0〉, |1〉, |0′〉, |1′〉} to Alice, which is the difference from J^+^11. The states |0′〉 = cos *θ*|0〉 + sin *θ*|1〉 and |1′〉 = sin *θ*|0〉 − cos *θ*|1〉, where 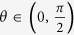. To perform a perfect detector-blinding attack as above, the relationship of equation (1) should be modified to


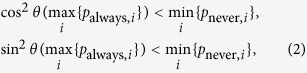


where cos^2^*θ* and sin^2^*θ* are the mutual projection probabilities of the honest states from different bases. Here, we take some specified values max_*i*_{*p*_always,*i*_} = 932 *μ*W and min_*i*_{*p*_never,*i*_} = 647 *μ*W for the simulation^26^. In this case, the range of *θ* that fulfils equation (2) is demonstrated in Fig. 1. We can see that if the selected value *θ* falls into the span of 0.586 ≤ *θ* ≤ 0.985, then dishonest Bob can launch the attack perfectly. However, when the *θ* is outside of the above range, Bob’s detector-blinding attack will introduce errors in Alice’s raw key. Note that 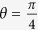 is the case for J^+^11, and the equation (1) is also satisfied.

From the above attack we can conclude that it is crucial to ensure that the experimental implementations of QKD-based QPQ are also secure. However, there is a gap between the theory and practice of QPQ in terms of the single-photon detectors, which dishonest Bob may exploit to break the user privacy without being detected. To solve this problem, the MDI-QPQ protocol is proposed in the following.

### Protocol of loss-tolerant MDI-QPQ

For the purpose of being completely loss independent, we demonstrate the polarization encoding MDI-QPQ protocol with single-photon sources.

**Figure 2:**
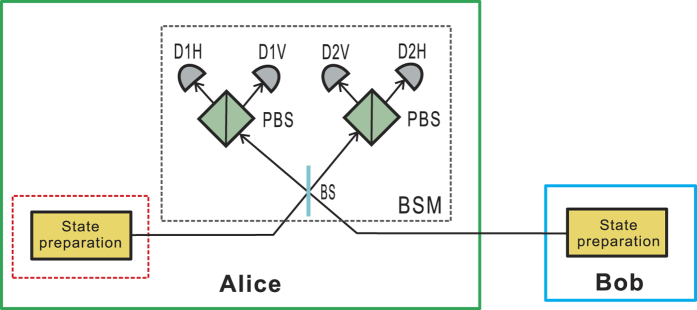
Schematic diagram of loss-tolerant MDI-QPQ for the honest parties. The BS represents 50:50 beam splitter, and PBS stands for polarization beam splitter. Alice and Bob prepare honest polarization states randomly with single-photon sources. Alice makes the BSMs. A joint click on D1H and D2V or D1V and D2H implies a projection into Bell state |Ψ^−^〉 and will be recorded by Alice. The red dotted box means that the classical information of Alice’s state preparation is not leaked to the BSM and Bob. The grey dotted box represents that Alice needs not to trust the BSM device.

(1) Bob sends, uniformly at random, one of the four polarized states |0〉, |1〉, and
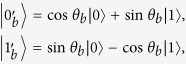
to Alice, where 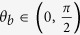 and can be adjusted to make the protocol have different degree of security. The rectilinear basis {|0〉, |1〉} encodes the raw key bit 0, and the basis 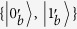 corresponds to the raw key bit 1.

(2) Alice prepares one of the four polarized states |0〉, |1〉, and
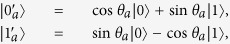
randomly and independently of Bob (the value of *θ*_*a*_ is related to *θ*_*b*_ and is calculated in next section in detail). Then, she projects her and Bob’s states into a Bell state (see Fig. 2). If the BSM does not output a Bell state, then Alice asks Bob to restart step (1). Otherwise, Alice records the measurement result. In fact, she needs to identify only the Bell state 
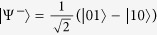
, which means that the BSM can be built with only linear optical elements. Theoretical probabilities of obtaining the projection |Ψ^−^〉 for different combinations of the honest states are shown in Table 1. Repeat steps (1) and (2) until *k* × *N* successful BSMs are made, where *k* is a natural number determined by the security analysis and *N* is the number of database’s bits. Note that this step will make the protocol completely independent of losses.

(3) For each trial Alice obtained a Bell state |Ψ^−^〉, Bob announces bit 0 to Alice if he has sent states |0〉 or 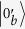, while reveals bit 1 if he has sent states |1〉 or 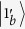.

(4) Alice decodes Bob’s states to acquire the corresponding raw key bits. It can be done depending on her state and the bit declared in step (3). The decoding process is given in next section specifically. Here, we take that the 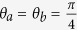 and Bob sends state |0〉 as an example. Now Bob will announce bit 0. According to Table 1, Alice can rule out 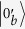 and conclude that the raw key is 0 certainly only if she prepared the state 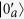. Both the conclusive and inconclusive raw key are retained.

(5) Now, a string of raw key with length *k* × *N* are distributed between Bob and Alice, where Bob knows every bit value and Alice knows partially. Then, Bob cuts the raw key into *N* substrings of length *k* and tells each bit’s position to Alice. The bits of every substring are added bitwise by Alice and Bob to form their final key of length *N*, respectively. For reducing the computation complexity of the error-correction procedure, if Alice is left with no final key bit composed by *k* conclusive raw key bits, henceforth referred to *query key* in this paper, then the protocol has to be restarted.

(6) Alice and Bob perform error correction on Alice’s final key using the method proposed by Chan *et al*.^18^. After that, Alice estimates the error rate of every query key. If all the error rates are less than some prescribed value, 10^−3^ for example^18^, then the protocol goes to the next step. Otherwise, the protocol aborts.

(7) Alice announces to Bob a shift value that will align one of the query key bits to the bit in the database she wants to retrieve. Then, Bob shifts his final key cyclically with this value, and uses the shifted key to encrypt his database by one-time-pad. Bob sends the encrypted database to Alice. Consequently, Alice can decode the queried item with that query key.

The security of MDI-QPQ is completely loss independent with single-photon sources. The reasons are as follows. First, we prove that the degree of insecurity for the database privacy is completely independent of the losses in practical system. Note that the four honest states are linearly dependent, implying that the unambiguous state discrimination (USD) is not applicable here^39^. Moreover, the density matrix of the state received by Alice in step (2) is


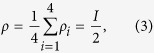


where *I* is the identity operator in two-dimensional Hilbert space, 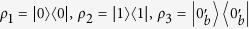, and 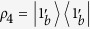. Equation (3) means that the identity operator can be resolved as a weighted sum over the density matrix *ρ*_*i*_, resulting that the maximal-confidence discrimination (MCD) of the four honest states can be done without inconclusive results^40^. Namely, the MCD coincides with the minimum-error discrimination (MED), and the maximal guessing probability cannot be increased by admitting inconclusive results. Therefore, in step (2), Alice cannot keep only the conclusive raw key by USD or MCD, and discard the inconclusive ones which can be attributed to the losses. On the other hand, thanks to the elegant design of SARG04 QKD, Alice still has to distinguish two non-orthogonal states even if she can store the photons and delay the measurements after step (3). If the distinguished result is uncertain, she has no chance to disregard it because this state has been declared detected successfully in step (2) and will be used for the following postprocessing. Consequently, Alice cannot know all the raw key, let alone the final key, no matter how many losses in the practical system.

As the BSM can be fabricated by a dishonest Bob, the measurement device can be designed to discriminate the four honest states randomly prepared by Alice, and announce the result to Bob through classical communication. The discrimination of Alice’s four honest states can be regarded as distinguishing the state *ρ* in equation (3) with 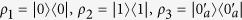, and 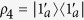. For the reasons explained above, Bob cannot resort to the USD and MCD to unambiguously distinguish Alice’s state. His optimal discrimination of Alice’s state is the MED. Thus, he would not determine the conclusiveness of Alice’s raw key with certainty. Namely, he will not acquire the position of the query key exactly at last. In a word, neither dishonest party will benefit from the losses in the practical implementation.

### Correctness

The degree of privacy for MDI-QPQ will be analyzed in three aspects. The first is the correctness of the protocol when both parties are honest. It can be seen from Table 1 that, to make the protocol work correctly, the absolute value of the difference between *θ*_*a*_ and *θ*_*b*_ should satisfies


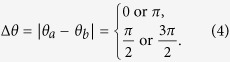


As there is no difference in the performance of the protocol for Δ*θ* = 0 and Δ*θ* = *π* (for 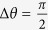 and 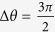 as well), we will choose Δ*θ* = 0 and 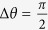 for demonstration. Now, the Table 1 becomes the Table 2.

We make an example to show how Alice obtains a conclusive raw key in the honest protocol. For Δ*θ* = 0, if Bob has sent state |0〉 and announced bit 0, according to Table 2, Alice can identify Bob’s state |0〉 and thus the raw key 0 with certainty only if she has prepared state 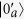. However, she will obtain an inconclusive result if she prepared states |1〉 or 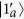. For 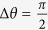, if Bob has sent state |0〉 and announced bit 0, according to Table 2, Alice can identify Bob’s state |0〉 and thus the raw key 0 with certainty only if she has prepared state 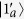. However, she will obtain an inconclusive result if she prepared states |1〉 or 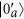.

As Alice selects the four states randomly, in the absence of noise, the probability that BSM yields the above conclusive raw key is


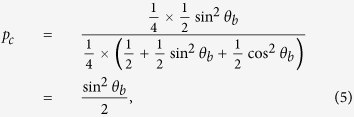


for both Δ*θ* = 0 and 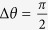. Without error correction, the average number of Alice’s query key is 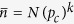. The probability that she will know none query key is *p*_0_ = [1 − (*p*_*c*_)^*k*^]^*N*^. It implies that the correctness of MDI-QPQ is the same with G^+^12 in the noiseless case. For databases with different number of bits *N*, we can choose suitable *k* and *θ*_*b*_ to make both the 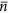 and *p*_0_ be small.

**Figure 3:**
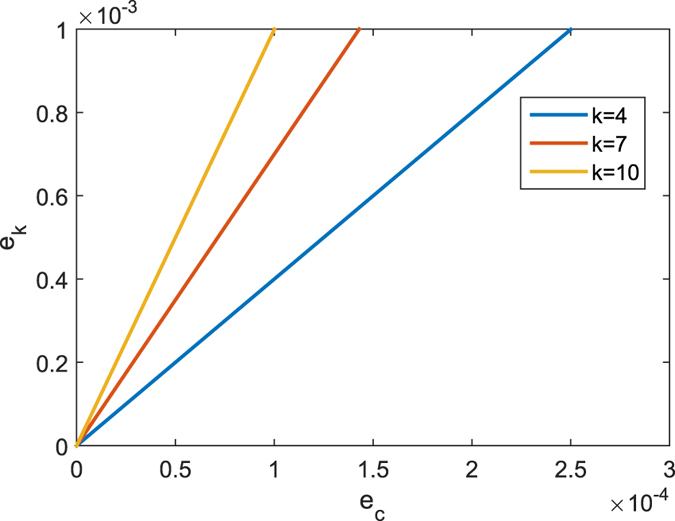
Relationships between the error rates *e*_*k*_ of the query key without error correction and *e*_*c*_ of the conclusive raw key for *k* = 4, *k* = 7 and *k* = 10 (from right to left).

In the presence of noise, the error rate of the query key before error correction is


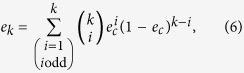


where *e*_*c*_ is the error rate of the conclusive raw key and odd errors happened in the the query key’s *k* conclusive raw key bits. The relationships between the error rates *e*_*k*_ and *e*_*c*_ for different *k* are shown in Fig. 3. It can be seen that even for very little noise, the error rate of the query key will increase to the threshold value 10^−3^ quickly. Thus, it is necessary to add the error-correction procedure^18^ into the protocol.

### Database privacy

Dishonest Alice will try to know as many query key as possible. Assume that Alice controls everything except Bob’s single-photon source. It is in Alice’s best interest to replace the noisy channel with a noiseless one to receive the states correctly each time. Although it is no matter that the channel is lossy, the all powerful dishonest Alice can use a lossless one instead. We also suppose she has perfect detectors and perfect quantum memories.

The imperfect privacy of database is guaranteed by the theorem that nonorthogonal quantum states cannot be distinguished perfectly^41^. And Alice must announce which states have been projected to Bell state |Ψ^−^〉 successfully before Bob tells some classical information of his sent states. Therefore, Alice inevitably obtains some inconclusive raw key with certain probability, which results in inconclusive final key.

As analyzed in J^+^11 and G^+^12 protocols^10^,^12^, Alice can increase the probability *p*_*c*_ by storing the received photons in quantum memories and performing the USD measurements after step (3). The successful probability of the USD, for Bob’s announced two honest states, is 
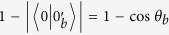
. Thus, the MDI-QPQ will obtain better database privacy for small *θ*_*b*_.

In this cheating strategy, Alice deviates from the MDI protocol to obtain more items of the database. However, our protocol is designed to protect only an honest Alice. Thus, it does not matter that the advantage of the MDI paradigm will not exist for a dishonest Alice.

### User privacy

Dishonest Bob will try to acquire the position of the item Alice queries. In the following, we will analyze the user privacy under the fact that Alice does not have to trust her measurement device. That is to say, we assume that the measurement device is built by dishonest Bob which contains his cheating equipment. Because the outcome of the measurement cannot reveal Alice’s state with certainty, the measurement device can send classical information to Bob. These are equivalent to that the measurement device is placed in Bob’s side and Bob announces the measurement results. Thus, all the detector side channels are removed. What Alice needs only to pay attention is to protect the classical information of her state preparation from leakage to the untrusted measurement device and Bob. Moreover, we suppose that both parties’ single-photon sources, the channels and the detectors are perfect. All these conditions will maximize the probability that Bob knows the item Alice queries.

#### Empty-pulse attack

Based on the above assumptions, we first introduce an *empty-pulse* attack in which Bob does not send any state in step (1). However, Bob places a cheating equipment in the measurement device to discriminate Alice’s states with minimum error rate. The measurement device will send the discrimination result to Bob and output a BSM projection randomly to honest Alice. Note that the four states sent randomly by Alice are pairs of two orthogonal states with the same *prior* probability 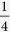. Thus, the guessing probability of the four honest states for the MED is 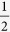
42. Namely, Bob’s discrimination has an error rate of 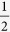.

For instance, assume Δ*θ* = 0 and the result of the MED is state |0〉. According to Table 2, Bob announces 0 in step (3) to expect Alice to acquire conclusive raw key 1. If the MED is right, Bob gains the information both on the conclusiveness and bit value of Alice’s raw key. However, if the MED is wrong, Alice will obtain conclusive key only if she prepares state 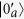. Now, Alice deems the raw key value is 0, which is different from Bob’s expectation. As Alice prepares the states randomly, in the empty-pulse attack the probability that Alice has conclusive raw key is


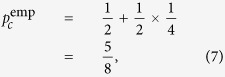


and the bit error rate of Bob’s corresponding raw key is 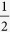.

#### Middle-state attack 1

Another more powerful attack is the *middle-state* attack proposed in the security analysis of J^+^11 and G^+^12 protocols^10^,^12^. In the middle-state attack on MDI-QPQ, Bob keeps the measurement device doing the BSM. However, instead of transmitting the honest states to Alice in step (1), he sends the middle state 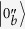 (or 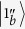) and announcing 1 (or 0) in step (3) to expect Alice to acquire a conclusive raw key. The forms of the two middle states are that


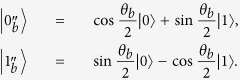


The theoretical probabilities for the middle states and Alice’s honest states being projected into Bell state |Ψ^−^〉 are shown in Table 3. Take the example that Bob sends state 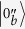 and announces bit 1. From Table 3 and the part of Table 2 for Δ*θ* = 0, it can be seen that Alice will obtain a conclusive raw key if she prepares states |1〉 or 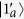. As for Δ*θ* = *π*/2, Alice will obtain a conclusive raw key if she prepares states |1〉 or 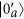. Because Alice prepares the states randomly, as shown in Table 3, the probability of obtaining a conclusive raw key in both cases is


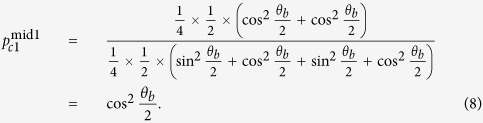


If Bob wants Alice to obtain an inconclusive raw key, he sends 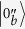 (or 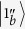) but announces 0 (or 1). Now, Alice will gets a conclusive result with probability


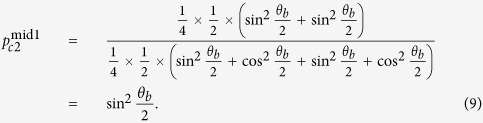


for both Δ*θ* = 0 and 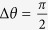.

**Figure 4:**
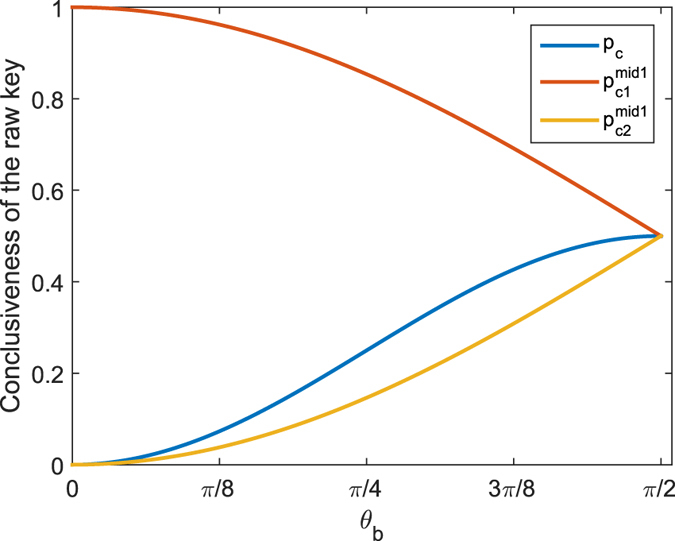
The probabilities of Alice acquiring a conclusive raw key in the honest protocol and in the first middle-state attack for different *θ*_*b*_. The curves represent 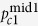, *p*_*c*_ and 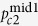 from up to down.

The relationships between *p*_*c*_, 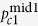, 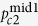 and *θ*_*b*_ are plotted in Fig. 4. We can see that Bob can increase or decrease the conclusiveness of Alice’s raw key in the middle-state attack. Thus, he will improve the accuracy of the estimation for Alice’s query address. However, it can be viewed from Tables 2 and 3 that now Alice will register the conclusive raw key bit value as 0 or 1 with equal probability, implying that the bit error rate of Bob’s corresponding raw key is 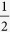.

#### Middle-state attack 2

There is another middle-state attack in which Bob can reduce the bit error rate of his raw key at the price of compromising his control of the conclusiveness of Alice’s raw key. The difference with the above one is that Bob now sends the following two middle states


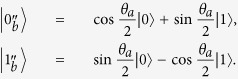


The theoretical probabilities for these two middle states and Alice’s honest states being projected into Bell state |Ψ^−^〉 are shown in Table 4. For the case Δ*θ* = 0, the two middle-state attacks are the same. In the following, we consider only the situation 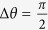 and assume the parties select 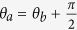. When Bob chooses the cheating strategy of sending the middle state 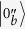 (or 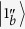) and announcing 1 (or 0), the probability which Alice obtains conclusive raw key is a constant


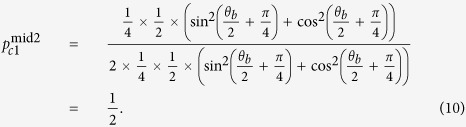


If Bob chooses sending 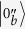 (or 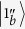) but announces 0 (or 1), Alice will gets a conclusive raw key with 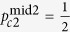 as well. It implies that the conclusiveness of Alice’s raw key is independent of Bob’s cheating strategies. Comparing with 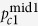 and 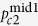, it shows that Bob has limited control over Alice’s raw key.

Take the cheating strategy that Bob sends the middle state 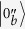 and announces 1 for instance. Alice will obtain a conclusive raw key if she prepares state |1〉 or 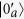. Seeing Table 4, the probability for states combination 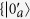 and 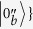 being projected into |Ψ^−^〉 is larger than the combination {|1〉 and 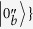. Thus, it is reasonable for Bob to guess that Alice has prepared state 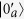 under the hypothesis that she obtained a conclusive result. If this is indeed the case, then Alice’s conclusive raw key bit is 0. However, if in fact, Alice prepares the state |1〉, she will identify the conclusive raw key bit 1 and Bob has a error in this raw key. Thus, the error rate of Bob’s raw key when compared with Alice’s conclusive results is


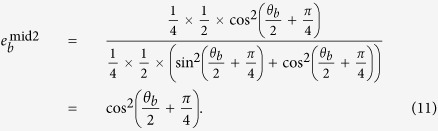


**Figure 5:**
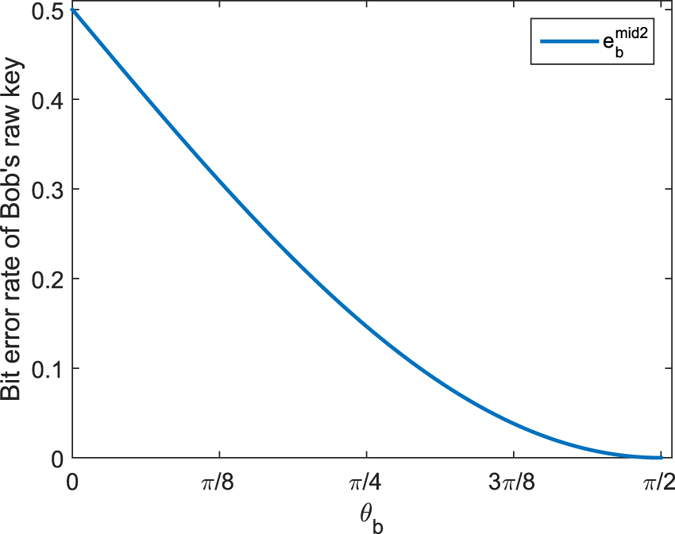
The relationship between the error rate of Bob’s raw key and *θ*_*b*_ in the second middle-state attack for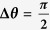.

The relationship between 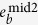 and *θ*_*b*_ is plotted in Fig. 5. It shows that if Bob pursues a low error rate of his raw key for 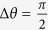, he can perform the second middle-state attack.

Comparing the two middle-state attacks, it shows that there is a trade-off between the control of the conclusiveness and knowing the value of Alice’s raw key. The first one allows Bob to obtain strong control on the conclusiveness of Alice’s raw key while makes him completely ignore of the key’s value. On the other hand, the second one let Bob know nonvanishing information on the value of the raw key, but his control on the key is limited.

In the above three attacks once Bob tries to gather more information on Alice’s query item than the honest protocol, it will make him loss the information on the raw key bit value, even he can control the measurement device. By designing the error-correction code properly, Bob’s final key is still not exactly the same with Alice’s corresponding query key at last^18^, which means that he may provide wrong answers. And it can be detected by Alice at a later time with a nonvanishing (but nonunity) probability. It shows that loss-tolerant MDI-QPQ is cheat sensitive for user privacy.

Actually, seeing Table 2, if Bob knows that one of Alice’s raw key is conclusive and its bit value is 0 (or 1) at the same time, then he can also correctly guess that the basis Alice used to prepare her state is 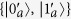 (or {|0〉, |1〉}) (note that in J^+^11 and G^+^12, Bob will correctly guess the basis Alice used to measure the state she received). However, since Alice has protected the classical information of her state preparation from leakage to Bob, the *no-signaling principle* implies that Bob’s probability to guess her basis correctly is no more than 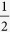. Thus, in MDI-QPQ, dishonest Bob cannot simultaneously have the bit value and the conclusiveness information of Alice’s raw key, which is similar to J^+^11 and G^+^12 protocols^10^,^12^.

## Discussion

We have proposed a detector-blinding attack launched by dishonest Bob on the practical QKD-based QPQ system. Specifically, perfect detector-blinding attack is always possible for practical J^+^11 system, and is possible for G^+^12 with a certain range of *θ*. However, the detector-blinding attack may introduce some errors in Alice’s raw key with smaller and larger *θ* value for G^+^12. To make the practical QKD-based QPQ systems secure again, we proposed the method of loss-tolerant MDI-QPQ. Compared with previous QKD-based QPQ, it has a distinct advantage of removing all the detector side channels. We have proven the security of loss-tolerant MDI-QPQ under some typical attacks. It would be meaningful to have a more general security analysis in the future. Moreover, the source flaws should be considered in the security analysis, because it has been assumed that both parties’ sources are trusted in this paper. We should examine this condition carefully in practice.

For the experiment of the proposed loss-tolerant MDI-QPQ, it can benefit from the rapid development of MDI-QKD experiments^28^,^29^,^30^,^31^,^32^,^33^,^34^,^35^ with only few necessary modifications. The major changes for the quantum process are the coefficients of the states prepared by both parties and they have to use single-photon sources. The remaining setups, like the BSM device, do not need to be modified. It shows that our proposal is feasible with the existed MDI-QKD experiments.

In the experiments of quantum cryptography, a PR-WCS source is always used to substitute the single-photon source, which is not easy for state-of-the-art technology. However, when we employ PR-WCS sources, the security of MDI-QPQ may be compromised due to the multi-photon pulses. Note that one could close the potential security loophole by limiting the total number of transmitted pulses, resulting that the protocol maybe not completely loss independent^43^. Another problem is that the projection |Ψ^−^〉 from the case when the parties prepare the states in bases 
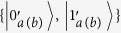
 will introduce a high inherent error rate for Alice’s raw key. Thus, an appropriate error-correction code is needed for the correctness of the protocol. Or one can refer the setup proposed for MDI-SARG04 QKD with PR-WCS sources^44^. We will deal with these obstacles in the following research to make the loss-tolerant MDI-QPQ more practical.

## Additional Information

**How to cite this article:** Zhao, L.-Y. *et al*. Loss-tolerant measurement-device-independent quantum private queries. *Sci. Rep.* **7**, 39733; doi: 10.1038/srep39733 (2017).

**Publisher's note:** Springer Nature remains neutral with regard to jurisdictional claims in published maps and institutional affiliations.

## Figures and Tables

**Table 1:** Theoretical probabilities of obtaining Bell state |Ψ^−^〉 for different combinations of the honest states.

(Combinations)	|Ψ^−^〉			
	|0〉	|1〉	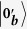	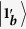
|0〉	0	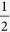	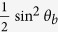	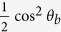
|1〉	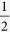	0	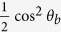	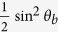
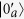	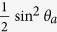	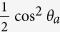	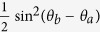	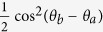
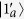	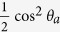	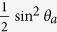	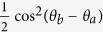	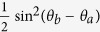

These can be calculated by the interferences of the honest states at the beam splitter.

**Table 2:** Theoretical probabilities of obtaining Bell state |Ψ^−^〉 for Δ*θ* = 0 and 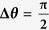 in the honest protocol.

(Δ*θ* = 0)	|Ψ^−^〉				|Ψ^−^〉				
	|0〉	|1〉	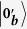	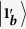	( 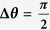 )	|0〉	|1〉	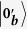	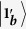
|0〉	0	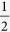	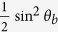	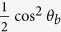	|0〉	0	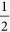	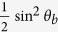	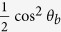
|1〉	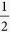	0	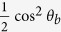	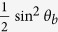	|1〉	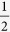	0	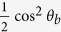	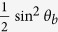
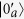	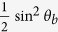	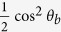	0	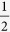	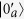	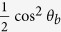	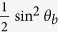	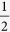	0
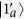	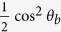	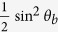	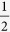	0	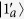	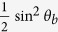	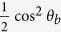	0	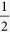

**Table 3:** Theoretical probabilities of obtaining Bell state |Ψ^−^〉 for different combinations of Bob’s cheating states and Alice’s honest states in the first middle-state attack.

(Δ*θ* = 0)	|Ψ^−^〉				|Ψ^−^〉				
	|0〉	|1〉	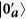	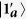	( 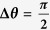 )	|0〉	|1〉	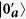	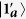
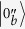	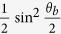	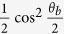	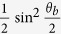	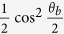	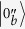	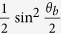	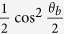	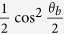	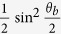
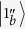	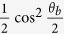	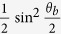	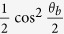	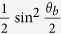	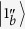	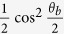	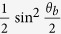	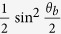	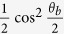

**Table 4:** Theoretical probabilities of obtaining Bell state |Ψ^−^〉 for different combinations of Bob’s cheating states and Alice’s honest states in the second middle-state attack.

(Δ*θ* = 0)	|Ψ^−^〉				|Ψ^−^〉				
	|0〉	|1〉	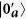	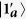	( 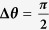 )	|0〉	|1〉	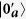	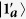
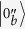	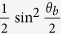	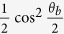	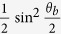	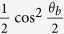	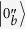	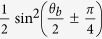	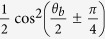	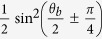	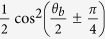
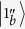	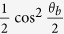	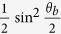	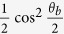	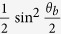	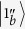	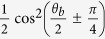	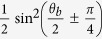	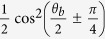	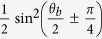

The operator ‘+’ in ‘±’ stands for the case 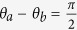, while the operator ‘−’ in ‘±’ represents the case 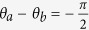.
